# An Isolated Greater Tuberosity Fracture With Posterior-Superior Displacement in an Elderly Patient: Successful Fixation Using Cannulated Screws With Washers Without Rotator Cuff Repair

**DOI:** 10.7759/cureus.91273

**Published:** 2025-08-30

**Authors:** Burak Ozturk, Muhammed Yusuf Afacan, Mohammadreza Hajizadeh, Cumhur Deniz Davulcu, Nuri Aydin

**Affiliations:** 1 Department of Orthopaedics and Traumatology, Istanbul University-Cerrahpasa, Cerrahpasa Faculty of Medicine, Istanbul, TUR; 2 Department of Anatomy, Istanbul University-Cerrahpasa, Institute of Graduate Studies, Istanbul, TUR

**Keywords:** conservative treatment, interfragmentary screw fixation, isolated greater tuberosity fracture, non-displaced fracture, posterior-superior displacement, proximal humerus fracture, rotator cuff injuries, rotator cuff repair surgery

## Abstract

Isolated fractures of the greater tuberosity are uncommon, comprising a minority of proximal humerus fractures. Their management depends primarily on the degree and direction of displacement. Posterior-superior displacement, in particular, is associated with subacromial impingement and compromised rotator cuff function, often necessitating surgical intervention.

This case report aims to present the management of an isolated greater tuberosity fracture with posterior-superior displacement in an elderly patient, emphasizing successful fixation with interfragmentary screws, without rotator cuff repair. We present the case of a 59-year-old female who sustained an isolated greater tuberosity fracture with significant posterior-superior displacement following an anterior shoulder dislocation. The patient was treated successfully with open reduction and internal fixation (ORIF) using cannulated screws with washers, without concurrent rotator cuff repair, as the tendons were intact, and anatomical reduction of the greater tuberosity fragment with screw fixation was sufficient to restore cuff function. Postoperative rehabilitation was initiated early, and the patient achieved full, pain-free range of motion and complete recovery of shoulder function within three months. The ASES (American Shoulder and Elbow Surgeons) and Constant scores were 90 and 88, respectively. This case demonstrates that interfragmentary screw fixation alone can yield excellent clinical outcomes in selected elderly patients, even in the absence of additional rotator cuff repair. Emphasis should be placed on accurate assessment of displacement via computed tomography (CT) imaging, and timely surgical intervention followed by structured rehabilitation.

## Introduction

The humerus is the principal long bone of the upper arm and plays a central role in shoulder articulation. Proximally, it consists of the humeral head, the anatomical and surgical necks, and the greater and lesser tuberosities [1]. These tuberosities act as critical attachment sites for the rotator cuff muscles: the subscapularis inserts onto the lesser tuberosity, while the supraspinatus, infraspinatus, and teres minor attach sequentially onto the greater tuberosity. Between them lies the intertubercular groove, which accommodates the tendon of the long head of the biceps, stabilized by the transverse humeral ligament and adjacent fibers of the subscapularis [1]. Proximal humerus fractures account for about 5% of all fractures and represent nearly half of the fractures involving the humerus [1]. Isolated greater tuberosity fractures represent a relatively uncommon subset of proximal humerus fractures, accounting for approximately 20% of all cases [1]. These fractures typically occur either as a result of direct trauma following a fall or as avulsion injuries secondary to shoulder dislocation [2]. These fractures complicate up to one-third of shoulder dislocations, particularly anterior shoulder dislocations [3]. For fractures without displacement or with minimal displacement (<2 mm), conservative treatment is usually sufficient. However, displaced fractures often require surgical intervention [4]. The patient's age, expectations, and the stability and displacement of the fracture fragment are influential factors in surgical decision-making [5]. Recent literature has indicated that surgical intervention may be warranted in greater tuberosity fractures with displacement as minimal as 3 mm, according to some authors [6]. While the primary factor influencing the treatment decision is the amount of fracture displacement, the literature highlights that assessments were made using computed tomography (CT). Fracture displacement was assessed on CT by orienting the humerus in coronal, sagittal, and axial planes, then measuring superior and posterior shifts at standardized points, with the greatest displacement used for analysis [5]. In these cases, 3D and 4D CT scans are valuable for accurately assessing fracture displacement [7].

Studies have reported dysfunction due to rotator cuff injury and subacromial impingement as complications of greater tuberosity fractures [8]. The main cause of movement restriction following a greater tuberosity fracture is the loss of rotator cuff function and subacromial impingement due to the posterosuperior displacement of the fracture [9]. The loss of rotator cuff function is simply due to the fact that its point of attachment has come off, and if it unites, it will have moved. The axillary nerve is the most frequently injured peripheral nerve of the shoulder, typically following glenohumeral dislocation, proximal humeral fracture, or direct trauma to the deltoid, and may also be affected in quadrilateral space syndrome, with unclear underlying mechanisms [10]. The treatment approaches and outcomes for isolated greater tuberosity fractures remain a topic of discussion in the current literature. In surgical treatment, patients may be treated with repair using anchors, double-row repair, interfragmentary screw fixation, or fixation with a locking plate. All surgical techniques may be performed using the deltopectoral approach. Interfragmentary screws are generally favored in younger male patients with good bone quality and split-type fractures, whereas suture anchors may be more appropriate in older female patients, or those with avulsion-type fractures and compromised bone stock [11].

The objective of this case report is to present the surgical management and clinical outcome of an isolated greater tuberosity fracture with posterior-superior displacement in an elderly patient, treated successfully with interfragmentary screw fixation without concurrent rotator cuff repair and to discuss its implications in the context of current literature.

## Case presentation

A 59-year-old female patient with a known history of major depressive disorder (quetiapine and sertraline) and no history of smoking presented to our clinic with right shoulder pain following a fall onto the shoulder. The patient reported that the trauma occurred two weeks earlier, when she fell onto her right shoulder from a standing height. She sought medical attention at an external facility abroad on the same day, where an anterior dislocation of the right glenohumeral joint was diagnosed. Reduction was achieved using a traction-countertraction maneuver, and a shoulder sling was applied there, as the patient described.

**Figure 1:**
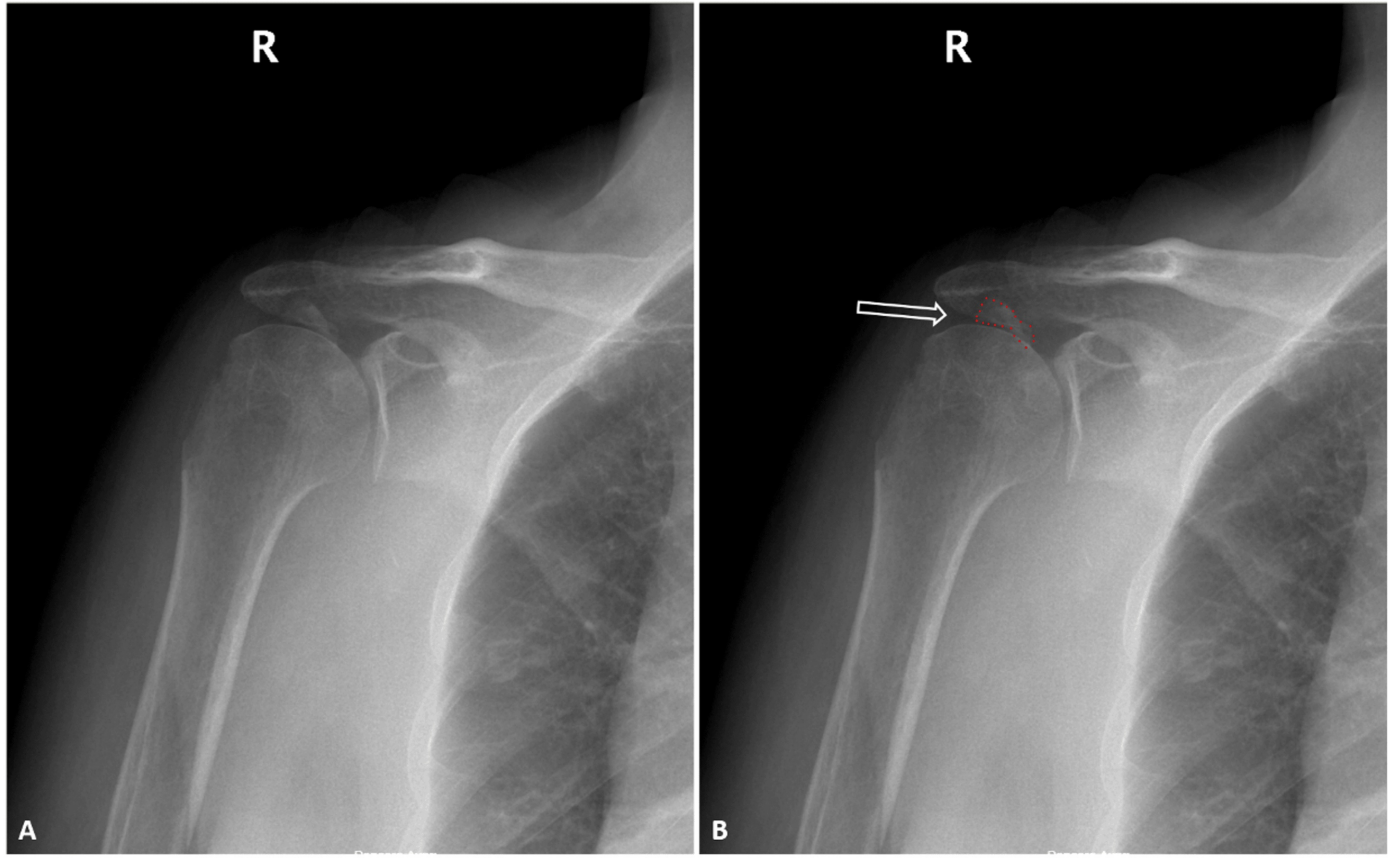
Patient’s Preoperative Radiographs Reveal a Fracture Involving the Right Greater Tuberosity True anteroposterior (A) radiograph of the right shoulder, with the white arrow and red circle in (B) indicating the displaced greater tuberosity fragment.

**Figure 2:**
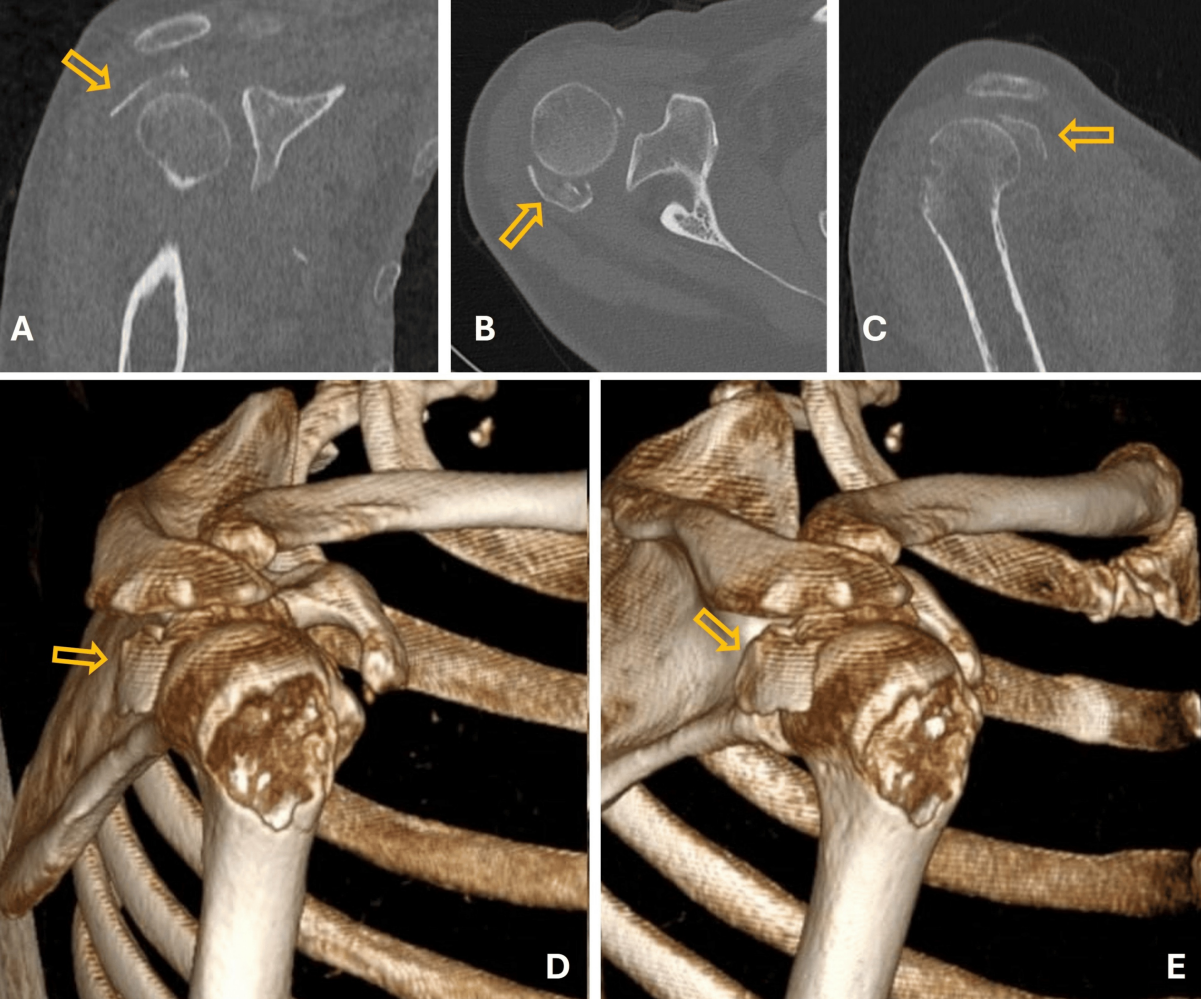
Computed Tomography (CT) and 3D Reconstruction Demonstrating Displaced Greater Tuberosity Fragment of the Right Shoulder Coronal (A), axial (B), and sagittal (C) CT images, along with three-dimensional reconstructions (D, E), demonstrate the displaced greater tuberosity fragment of the right shoulder (yellow arrows).

**Figure 3:**
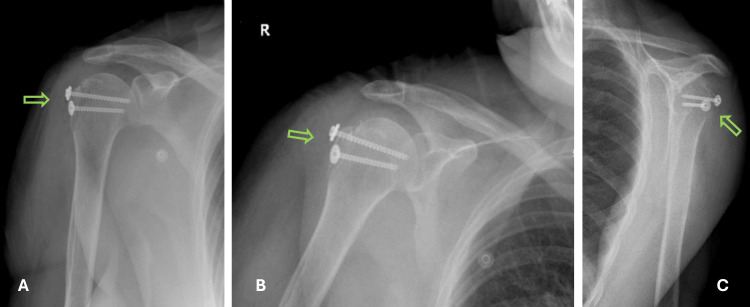
Postoperative Radiographs Demonstrating Open Reduction and Internal Fixation of the Greater Tuberosity Fragment With Cannulated Screws and Washers Postoperative true anteroposterior (A), anteroposterior (B), and scapular Y (C) radiographs of the right shoulder, demonstrating open reduction and internal fixation of the greater tuberosity fragment with two cannulated screws with washers (green arrows).

On physical examination in our clinic, the patient had extensive swelling and bruising in the right shoulder region. The range of motion in her right shoulder was limited, and she had tenderness over the proximal humerus on palpation. Neurovascular examination revealed no abnormalities. Radiological evaluations, including shoulder X-rays (Figure 1) and a non-contrast thin-slice 3D CT scan (Figure 2), revealed an isolated greater tuberosity fracture with posterosuperior displacement of the fracture fragment. The displacement measured 39 mm. The patient was treated with open reduction and internal fixation (ORIF) using cannulated screws with washers through a deltoid-splitting approach (Figure 3). 

Postoperatively, a shoulder sling without a pillow was applied to prevent further displacement of the fracture. The patient was followed weekly during the first two weeks, and then at two-week intervals. On the night of the surgery, passive elbow and pendulum exercises were initiated, and the patient subsequently continued pendulum exercises four times a day, with 20 repetitions per session. These consisted of the arm being released downward with gravity in lumbar flexion, allowing free oscillatory movements in forward-backward, side-to-side, and circular directions, both clockwise and counterclockwise. From the fourth week onward, wand exercises were introduced to enhance the shoulder joint range of motion, including wall-climbing movements in which the patient used the fingers to stepwise walk up the wall in order to gradually increase forward elevation. By the sixth week, the patient's shoulder pain had completely resolved, and a rotator cuff strengthening program was commenced. This program included targeted exercises such as resisted abduction in the scapular plane to strengthen the supraspinatus; theraband-assisted or manual resistance external rotation exercises to reinforce the infraspinatus and teres minor; and scapular retraction and shrugging exercises to enhance trapezius function and scapular stabilization. By the third- to sixth-month and one- to two-year evaluations, the strength of the right rotator cuff muscles was rated 5/5, the patient had a pain-free range of motion of 180°, and the ASES (American Shoulder and Elbow Surgeons) and Constant scores were 90 and 88, respectively.

## Discussion

Greater tuberosity fractures are more frequently encountered as part of complex proximal humerus fractures, while their occurrence as isolated injuries is relatively uncommon. Isolated greater tuberosity lesions constitute only 14% to 20% of all proximal humerus fractures [4]. Approximately 30% of greater tuberosity fractures are reported in association with anterior shoulder dislocations [12].

According to Neer, a displacement of one fragment greater than 10 mm and 45° is considered an indication for operative treatment [13]. The degree of fracture displacement remains a matter of debate, particularly due to limitations in measurement techniques. When assessed using only plain radiographs, discrepancies as large as 13 mm have been documented [14]. Research has demonstrated that the choice of imaging modality does not influence the reliability of fracture evaluation or the decision for surgical intervention [15]. Although discrepancies such as underestimation of displacement by plain radiographs have been reported, overall treatment decisions may remain unaffected, as the clinical threshold for surgical indication is generally maintained.

The current literature lacks robust evidence favoring either conservative or surgical treatment approaches for greater tuberosity fractures. It remains uncertain whether factors such as fracture type and underlying etiology significantly impact clinical decision-making or treatment outcomes. Notably, data on the nonoperative management of isolated greater tuberosity fractures are particularly limited.

One study demonstrated that interfragmentary screw fixation provided superior biomechanical results in young male patients with adequate bone quality and displaced fracture patterns [11]. In the present case, however, the patient was elderly, and despite literature recommendations favoring the use of suture anchors in such cases [11], we opted for interfragmentary screw fixation with washers to unite the greater tuberosity and proximal humerus. Surgical treatment is generally indicated in isolated greater tuberosity fractures when displacement exceeds 5 mm [4], and in our case, this threshold was surpassed, with 39 mm of displacement, justifying surgical intervention. Thus, although our fixation method differed from the conventional recommendation for elderly patients, the decision to proceed with surgery was consistent with the indications reported in the literature [4,11].

In a study conducted by Platzer et al. in 2008, they compared the outcomes of 52 patients with greater than 5 mm displacement who underwent ORIF with nine similar patients who were treated conservatively [16]. Surgical techniques included ORIF via a deltoid-splitting approach using sutures or tension band wiring, and closed reduction with percutaneous or minimally invasive fixation (CRPF) using cannulated lag screws [16]. After an average follow-up of five years, they concluded that functional outcomes, as assessed by the Constant Score, the Vienna Shoulder Score (VSS), and the University of California, Los Angeles (UCLA) Score, were significantly better following operative treatment [16].

The same authors reported on 135 patients with isolated greater tuberosity fractures treated conservatively and found that fractures with displacement greater than 3 mm had slightly inferior functional outcomes, with a mean Constant Score of 75, a VSS of 9.2, and a UCLA Score of 28.7, compared to higher values in those with less displacement [4].

In isolated greater tuberosity fractures, fragment displacement - particularly in the posterosuperior direction - is associated with impaired function and worse outcomes, as reflected by Constant-Murley and DASH (Disabilities of the Arm, Shoulder, and Hand) scores [17]. In another study, a professional young swimmer with a greater tuberosity fracture-dislocation of less than 5 mm displacement was successfully managed non-surgically, achieving full shoulder range of motion, despite it being his first dislocation and the initial suspicion of an anterior labroligamentous periosteal sleeve avulsion (ALPSA) lesion [18].

In this case, we especially preferred screws with washers because our patient was elderly and had reduced bone quality, where additional support was essential to maintain fixation integrity. Washers were used in conjunction with the cannulated screws to enhance fixation stability by distributing compressive forces over a wider bony surface. This reduces the risk of screw head penetration, cut-out, or fragment comminution - particularly in osteoporotic bone or small fracture fragments - thereby ensuring secure fixation and promoting reliable fracture healing [19].

## Conclusions

Isolated greater tuberosity fractures are uncommon, and the degree of fragment displacement remains the most critical factor in determining the appropriate treatment approach. Plain radiographs may underestimate displacement due to superimposition, whereas 3D and, if possible, 4D CT imaging provides a more accurate assessment. Posterior-superior displacement is particularly associated with impaired shoulder function and poorer outcomes due to its impact on rotator cuff integrity. In our case, the presence of significant posterior-superior displacement and symptomatic impingement warranted surgical intervention, resulting in excellent clinical and radiological outcomes without the need for additional rotator cuff repair. Although the literature recommends double-row anchor fixation, especially in older female patients, our experience demonstrates that interfragmentary screw fixation of the greater tuberosity with the proximal humerus can also achieve successful union and full joint mobility in such cases. Ultimately, the key to successful management may lie not only in the surgical technique but also in the timely decision for operative treatment and the implementation of early rehabilitation protocols. As this is a single case report, the findings should be interpreted with caution; nonetheless, it contributes to the limited but growing clinical experience on the management of isolated greater tuberosity fractures.
